# Modulation of Malaria Phenotypes by Pyruvate Kinase (*PKLR*) Variants in a Thai Population

**DOI:** 10.1371/journal.pone.0144555

**Published:** 2015-12-14

**Authors:** Rebekah van Bruggen, Christian Gualtieri, Alexandra Iliescu, Chalisa Louicharoen Cheepsunthorn, Punchalee Mungkalasut, Jean-François Trape, David Modiano, Bienvenu Sodiomon Sirima, Pratap Singhasivanon, Mark Lathrop, Anavaj Sakuntabhai, Jean-François Bureau, Philippe Gros

**Affiliations:** 1 Department of Human Genetics, Faculty of Medicine, McGill University, Montreal, Quebec, Canada; 2 Department of Biochemistry, Faculty of Medicine, McGill University, Montreal, Quebec, Canada; 3 Department of Biochemistry, Faculty of Medicine, Chulalongkorn University, Bangkok, Thailand, 10330; 4 Laboratoire de Paludologie et Zoologie Médicale, Institut de Recherche pour le Développement, Dakar, Sénégal; 5 Department of Public Health and Infectious Diseases, Instituto Pasteur-Fondazione Cenci Bolognetti, Sapienza University of Rome, Rome, Italy; 6 Centre National de Recherche et de Formation sur le Paludisme, Ministry of Health, Ouagadougou, Burkina Faso; 7 Department of Tropical Hygiene (Biomedical and Health Informatics), Faculty of Tropical Medicine, Mahidol University, Bangkok, Thailand; 8 Unité de la Génétique Fonctionnelle des Maladies Infectieuses, Institut Pasteur, Paris, France; 9 Centre National de la Recherche Scientifique, URA3012, F-75015, Paris, France; Liverpool School of Tropical Medicine, UNITED KINGDOM

## Abstract

Pyruvate kinase (PKLR) is a critical erythrocyte enzyme that is required for glycolysis and production of ATP. We have shown that *Pklr* deficiency in mice reduces the severity (reduced parasitemia, increased survival) of blood stage malaria induced by infection with *Plasmodium chabaudi* AS. Likewise, studies in human erythrocytes infected *ex vivo* with *P*. *falciparum* show that presence of host PK-deficiency alleles reduces infection phenotypes. We have characterized the genetic diversity of the *PKLR* gene, including haplotype structure and presence of rare coding variants in two populations from malaria endemic areas of Thailand and Senegal. We investigated the effect of *PKLR* genotypes on rich longitudinal datasets including haematological and malaria-associated phenotypes. A coding and possibly damaging variant (R41Q) was identified in the Thai population with a minor allele frequency of ~4.7%. Arginine 41 (R41) is highly conserved in the pyruvate kinase family and its substitution to Glutamine (R41Q) affects protein stability. Heterozygosity for R41Q is shown to be associated with a significant reduction in the number of attacks with *Plasmodium falciparum*, while correlating with an increased number of *Plasmodium vivax* infections. These results strongly suggest that *PKLR* protein variants may affect the frequency, and the intensity of malaria episodes induced by different *Plasmodium* parasites in humans living in areas of endemic malaria.

## Introduction

Malaria is one of the clearest examples of host genetic contributions to susceptibility to infections (reviewed in [1–4]). Indeed, the number of clinical episodes of malaria, the level of blood parasitemia during infection, the rate of transmission (gametogenesis), and the severity of disease developed (mild, severe malaria-induced anemia, cerebral malaria), all show a strong heritable component [1, 2, 5–10]. The complexity and nature of the genetic factors regulating these traits have been studied in case-control studies with candidate genes and in a few family-based genome wide linkage analyses [1, 2, 11–14]. Genetic variants affecting invasion of erythrocytes by merozoites, intra-erythrocytic replication or elimination of parasitized RBC have a major effect on infection. For example, the Duffy antigen is the receptor for *P*. *vivax* on erythrocytes and its absence in the Duffy negative blood group prevents parasite entry in erythrocytes and protects against *P*. *vivax* malaria [12, 13]. Glycophorins (GYPA, GYPB, GYPC) bind to *Plasmodium* surface proteins, and GYPC-non expressing individuals show reduced invasion of erythrocytes, and are protected from infection [14]. Deletion of the anion exchanger Band 3 protein causes Melanesian ovalocytosis, which is also linked to reduced malaria incidence [15]. Heterozygosity for mutant haemoglobin (Hb) variants causing either sickle cell anemia (HbS) [16, 17] or thalassemias [4, 18–20] provide significant protection against malaria, with strong positive selection of mutant alleles in malaria-endemic areas. Glucose-6-phosphate dehydrogenase (G6PD) is required for glutathione production and protection against Hb degradation-induced oxidative stress damage. G6PD deficiency offers very strong protection against *P*. *vivax* but not against *P*. *falciparum* malaria [11]. Finally, A/B blood group antigens contribute to rosetting of parasitized RBCs, and a recent large population study has identified diminished risk of malaria in the O blood group [21, 22].

Pyruvate kinase (PK) catalyzes the last rate-limiting step of glycolysis. There are two genes in humans that code for pyruvate kinases, the liver/erythrocyte-specific enzyme (PKLR) and the muscle specific enzyme (PKM1/2). In mature erythrocytes, PKLR is essential for energy generation [23]. PKLR is active as a tetramer, removing the phosphate from phosphoenolpyruvate (PEP), and producing pyruvate and ATP [23–27]. PK-deficiency (OMIM#266200) is the most common cause of non-spherocytic hemolytic anemia, and is inherited in an autosomal recessive manner [24, 28, 29]. Clinical manifestation of PK-deficiency in humans ranges from mild compensated hemolytic anemia to severe hemolysis causing neonatal death [23, 24, 29–31]. In mice, Pklr-deficient strains AcB55 and AcB61 (Pklr^I90N^) and CBA/Pk^slc^ (Pklr^G338D^) display haemolytic anemia caused by homozygosity for loss of function mutations in the Pklr enzyme. Following infection with *P*. *chabaudi* AS, these Pklr-deficient mice show decreased peak parasitemia and decreased mortality [31, 32]. On the other hand, we have previously shown that erythrocytes from PKLR-deficient human patients are significantly less permissive to *P*. *falciparum* infection than PKLR-sufficient erythrocytes [30]. In addition, erythrocytes from heterozygous PKLR-deficient patients parasitized with *P*. *falciparum* are phagocytised at appreciably greater levels than control parasitized erythrocytes [30, 33]. Therefore, PKLR-deficiency in humans is associated *ex vivo* with reduced erythrocyte invasion and increased phagocytosis of early-stage infected erythrocytes.

Sequencing the *PKLR* gene from 387 normal individuals from different regions of the world including areas where malaria is endemic (CEPH Human Diversity Panel) showed that the Sub-Sahara African cohort exhibited the highest genetic diversity within *PKLR*, while the European population exhibited the lowest level of genetic diversity [34]. These findings were replicated in another study, suggesting that the *PKLR* gene has been under selective pressure, possibly due to malaria [35]. On the other hand, case-control studies have not detected association of *PKLR* variants with malarial phenotypes [35, 36]. However, disease-based cohorts with single clinical end-points (presence or absence of disease) are not optimal to test the potential protective effects of *PKLR* variants on human malaria. In such cohorts, it is difficult to distinguish individuals with ‘temporal’ resistance (non-infected, infected but asymptomatic, or displaying sub-clinical phenotypes at the time of sampling) from those with true resistance, whereby these individuals are infected but do not develop disease (severe or otherwise) [8]. These issues can be best addressed in longitudinal studies of community-based cohorts, wherein different quantitative malarial phenotypes such as fever, parasitemia, type and number of malaria episodes are studied over time in the same individuals. Genetic studies in these unique cohorts have increased sensitivity and can detect genetic effects on different discrete disease endophenotypes [8, 37, 38]. In this study, we took advantage of two longitudinal population studies conducted in the malaria endemic areas of Senegal and Thailand, to identify whether *PKLR* gene variants are associated with protection against the incidence or severity of malarial infections.

## Materials and Methods

### Population Studies

The population cohorts and associated longitudinal population studies in the Senegalese villages of Dielmo and Ndiop (n = 878) [7, 8, 39] and the studied Thai population (n = 897) [6, 7, 11] have been previously described. Passive and active surveillance for identification of clinical and subclinical phenotypes has been described [6–8, 11, 39–41]. Human DNA samples were obtained with written informed consent, and were approved by the ethical review Committee for Research in Human Subjects, Ministry of Public Health, Thailand. In Senegal, the project was approved by the Ministry of Health of Senegal, and by the village population. Approval is renewed on a yearly basis. Written informed consent was obtained from all participants over the age of 15 years or from the guardians of children younger than 15 year. In all cases, such consent was obtained in the presence of the school director, an independent witness. Written consent was recorded on a voluntary consent form written in both French and Wolof, the local language. This methodology was approved by the National Ethics Committee of Senegal, which carries out audits regularly along with *ad hoc* committees of the Ministry of Health, the Pasteur Institute (Dakar, Senegal) and the Institut de Recherche pour le Développement (Marseille, France). Patients’ records and information were de-identified prior to analysis.

Subsets of these populations were investigated for variants in *PKLR*, using different size exploratory and validation cohorts (see Results). Exons and exon/intron junctions of the *PKLR* gene were sequenced using primer pairs adapted from [34] (S1 Table). Sequences were aligned to the *PKLR* gene (chr1:155259084–155270792) from the hg19 (Feb 2009) human assembly (UCSC) using BioEdit Sequence Alignment Editor [42]. Haploview software [43] was used to identify haplotypes for each community using common dbSNPs (MAF≥10%). Using Custom Taqman SNP primers and probes designed for rs147659527 (L272V) and R41Q, the presence of these variants were analyzed within the Senegalese and Thai communities, respectively. In addition, we examined the R41Q variant in 340 unrelated individuals from six Southeast Asian populations (52 Mon, 62 Burmese, 65 Cambodian, 104 Thai, and 57 Lao).

### Association studies and statistical analysis

The rs147659527 (L272V) genotypes were determined using custom Taqman SNP primers. The R41Q genotypes were determined using a high-resolution melting test with forward primer 5’-AAGGGCAGGTGACATGCAGT -3’ and reverse primer 5’-TGCTGCTGGAAGAAGGCAGT-3’, and analyzed with a CFX96 Touch Real-Time PCR Detection System (BIO-RAD). HRM genotypes were confirmed by PCR-RFLP, and by direct sequencing of PCR products. When possible, familial segregation of genotypes was verified using the Pedcheck software [44]. In the Thai cohort (S2 Table), we tested association of the R41Q variant with 37 blood cell counts and associated haematological parameters, as well as with 9 malaria-related phenotypes as described [6] (listed in S3 Table). All malaria-related phenotypes were analyzed for R41Q genotypes and no associations were detected except for the two phenotypes, the *Plasmodium falciparum* attacks and *Plasmodium vivax* attacks. There is no trait defining the “severity” of malaria *per* se in this hypo-endemic area. Attacks are defined as measured fever (axillary temperature > 37.5°C) or fever-related symptoms including headache, back pain, chills, myalgia, nausea and vomiting, associated with a blood smear positive for blood-stage trophozoite *P*. *falciparum* or *P*. *vivax*. The delay between two attacks with the same parasite is at least 30 days. For the two attack phenotypes, a Generalized Linear Mixed Model was fitted with the individual person as a factor in the random model. A Poisson error structure was implemented, thus yielding a log-linear regression. “Explanatory factors” include year (1998–2005), month, hamlet, and gender, with age factored into eight groups. The Pearson residual variance, not explained by “environmental” factors, was generated for each attack, and the sum of the residuals per person and for each parasite was used as the attack phenotype. R41Q heterozygotes were compared with R41 wild-type homozygotes for each trait using a parametric ANOVA test, taking into account normal variables affecting these traits. For non-Gaussian distribution, the non-parametric Kruskal-Wallis equality-of-populations rank test with one degree of freedom was also utilized. Analysis was completed first on non-stratified and then on gender-stratified samples. For ANOVA, mean ± SEM and the number of individuals were given for each group.

### Preparation of wild-type and mutant *PKLR* constructs

Wild-type (WT) *PKLR* cDNA in a pET-21a vector was generously provided by Dr. G. Valentini (University of Pavia, Italy). An NheI restriction site (5’-G^CTAGC), Kozak consensus sequence (5’-CCACC) and an HA epitope tag (5’-TACCCATACGATGTTCCAGATTACGCT) were added to the N-terminus, while a (His)_6_ tag (5’-ATGATGATGATGATGATG) was added at the C terminus via PCR amplification using primer pairs (5'-GTG CTA GCC CAC CAT GTA CCC ATA CGA TGT TCC AGA TTA CGC TTC GAT CCA GGA GAA CAT ATC ATC C -3’ and 5'-TAA TGT TCT AGA TCA ATG ATG ATG ATG ATG ATG GGA TAT GCT TAG CAC CCG CAT GA -3’). The WT construct was inserted into the pcDNA3 vector and used as template for the introduction of mutations by site-directed mutagenesis (QuikChange site-directed mutagenesis kit, Stratagene) using mutation primers (S4 Table). The veracity of mutant variants was verified by DNA sequencing (S5 Table).

### Protein Expression Studies

Twenty-four hours prior to transfection, 5 x 10^5^ HEK293T cells/well were plated in complete media [DMEM (Wisent Inc., St. Bruno, QC) + 10% FBS (Wisent, Inc.) + penicillin/streptomycin (ThermoFisher Scientific, South Logan, Utah)] in 6-well tissue culture dishes. Recombinant *PKLR* construct DNA was mixed with Opti-MEM (Life Technologies) and Lipofectamine 2000 (Invitrogen, Carlsbad, CA) prior to being added to cells supplemented with fresh DMEM + 10% FBS. Transiently transfected cells were harvested 18 hrs later. In some experiments, transfected cells were plated in complete medium supplemented with 0.5mg/mL Geneticin (G418, Gibco Life technologies, Grand Island, NY), and G418-resistant colonies were isolated 14 days later. Transfected cells were washed in 1xPBS (HyClone, Logan, Utah), then sonicated in PBS (6x15sec) on ice (Vibra-cell Sonicator, Sonics and Materials Inc., Newtown, CT). The total protein concentration within the cell lysate was quantified using a Bradford assay (Bio-Rad Laboratories, USA), and equal amounts of protein lysate were separated on a 10% SDS-PAGE gel, followed by immunoblotting against HA.11 Clone 16B12 purified monoclonal antibody (mouse) (Covance Inc., Princeton, NJ) and β-actin antibody (mouse) (Covance Inc.) as a loading control. The secondary antibody, ECL^TM^ Anti-mouse IgG, Horseradish Peroxidase linked whole Ab (from sheep) (GE Healthcare Ltd., Buckinghamshire, UK), was used in conjunction with SuperSignal WestPico Chemiluminescent substrate (ThermoFisher Scientific) for protein detection.

### Protein Stability Assay

For protein stability assays, cells were incubated in methionine- and cysteine-free DMEM media containing 10% dialyzed FBS, and 55 μCi of [^35^S] methionine-cysteine (Perkin-Elmer, Boston, MA). Cells were then washed twice with PBS and incubated in chase medium (standard DMEM containing 10% FBS, 100 μg/ml methionine and 100 μg/ml cysteine) for up to 24 hours. For immunoprecipitation, equal amounts of cell lysates were incubated with the mouse anti-HA antibody overnight at 4°C, followed by incubation with protein A/G-sepharose (GE Healthcare, Piscataway, NJ) for 4 hrs. Beads were washed three times in RIPA buffer and proteins were eluted with 50μl of 3X Laemmli sample buffer. The radiolabeled proteins were separated by electrophoresis using 7.5% SDS polyacrylamide gels. The gels were then fixed, impregnated with Amplify (GE Healthcare, Piscataway, NJ), dried and exposed to film. Densitometric analysis was performed using NIH ImageJ software (NIH, Bethesda, MD).

### Mice and parasite

CBA/Pk^slc^ mice were provided by the Japan SLC Animal Facility (Mr. H. Asai) and subsequently maintained as a breeding colony at McGill University. CBA/CaHn-Btk/J mice were purchased from Jackson Laboratories (Bar Harbor, ME, USA). All mice were kept under specific pathogen free conditions and handled according to the guidelines and regulations of the Canadian Council on Animal Care. Mice experimentation protocol was approved by the McGill Facility Animal Care Committee (P. Gros, Principal Investigator; protocol number: 5287), and include procedures to minimize distress and improve welfare. An LDH virus-free isolate of *Plasmodium chabaudi* AS (D. Walliker; University of Edinburgh) was maintained by weekly passage in A/J mice by intra-peritoneal infection with 10^6^ parasitized erythrocytes (pRBCs) [45]. For blood-stage infections, 8–12 weeks old mice were infected *i*.*v*. with 10^5^ pRBCs suspended in pyrogen-free PBS; blood parasitemia was monitored daily (days 4–28) on thin blood smears stained with Diff-Quik (Dade Behring). Mice infected with *Plasmodium chabaudi* were monitored twice daily for appearance of malaria symptoms at the peak of blood stage replication. Animals showing lethargic behavior and/or ruffled fur appearance or significant weight loss were sacrificed following anesthesia.

## Results

### *PKLR* genotypes in informative Senegalese and Thai populations

We investigated three populations living in areas of endemic malaria (Senegal; Thailand), and in which several haematological and malarial phenotypes were followed longitudinally [6–8, 11, 39]. In these populations, we tested the hypothesis that coding or non-coding variants in the *PKLR* gene may be associated with differential response to the malarial parasite, including episodes and intensity of infections. As a first step, we sequenced exons and intron/exon junctions of the *PKLR* gene in an exploratory cohort of 62 individuals from Senegal (35 from Dielmo, 27 from Ndiop) and 6 individuals from the Thai-Myanmar border region.

**Fig 1:**
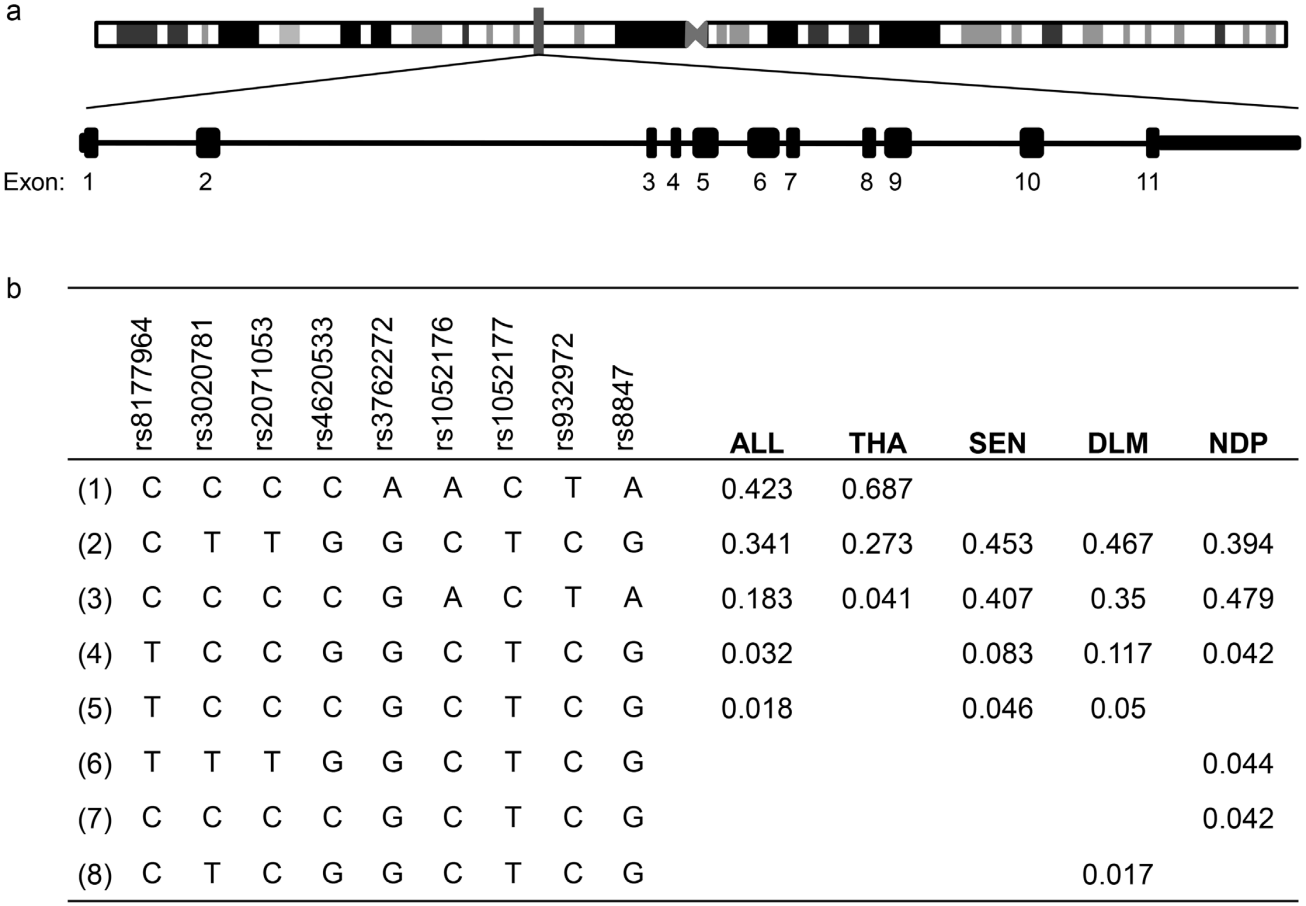
PKLR gene variants discovered in both the Senegalese and Thai community. **(a)** Location of coding and non-coding variants within the *PKLR* gene. Encoded on chromosome 1q22 (155259084–155270792 base pairs), the erythrocyte isoform, transcribed from a tissue specific promoter has 11 exons (blocks). **(b)** Haplotypes and haplotype frequencies for sequenced population. Determined using the Haploview software, haplotypes were defined by the SNPs with a MAF ≥ 10% that defined the Senegalese and Thai populations. Seven of the SNPs were shared within the three study populations, while rs4620533 was specific for the Senegalese populations and rs3762272 was found only within the Thai population. Haplotype frequency distribution were calculated for the complete data set (ALL), or subdivided on country (Thailand, THA; Senegal, SEN) or village (Dielmo, DLM; Ndiop, NDP).

Nineteen single nucleotide polymorphisms (SNPs) were identified in the Senegalese communities (Table 1), including several known dbSNPs (MAF>10%) as well as a number of rarer variants. Of note, rs147659527 (c.853C>G) causes a conservative L272V substitution identified in a single homozygous Ndiop individual (Table 1). Upon further investigation, this individual did not display symptoms of PK-deficiency, suggesting that L272V does not affect PKLR protein activity and/or is phenotypically neutral. *PKLR* haplotypes were defined using eight SNPs with MAF>10% (rs8177964, rs3020781, rs2071053, rs4620533, rs1052176, rs1052177, rs932972, and rs8847). The Dielmo and Ndiop communities displayed five haplotypes each, three of which were shared between them, with the remaining less frequent haplotypes being unique to each community (Fig 1).

**Table 1:** Single nucleotide polymorphisms and alternative allele frequencies within the sequenced population.

					Senegal		Thailand	
SNP	Position	Coding	Ref	Alt	Dielmo % (Total)	Ndiop % (Total)	Combined % (Total)	dbSNP build 138 (%)
rs187698692	155270961		C	T	1.43 (35)			0.05
rs149505733	155270855		G	A			1.58 (95)	0.14
SNP1	155270758		A	G	1.43 (35)			
R41Q	155270050	R41Q	G	A			4.90 (102)	
rs8177962	155269991	L61L	C	T	1.43 (35)			0.53
rs8177963	155269830		T	A	10.00 (35)	3.85 (26)		1.28
rs8177964**	155269780		C	T	14.29 (35)	7.69 (26)		3.12
rs3020781**	155269776		C	T	54.29 (35)	42.31 (26)	28.92 (102)	49.30
K105K	155265516	K105K	G	A		1.85 (27)		
rs2071053**	155265177		T	C	47.14 (35)	59.26 (27)	72.11 (95)	45.75
SNP2	155264607		C	G	1.47 (34)			
rs200695047	155264433	V269F	G	T			4.08 (98)	0.09
rs147659527	155264424	L272V	C	G		3.85 (26)		0.16
rs8177979	155263430		C	T	4.29 (35)	3.70 (27)		1.70
SNP3	155263186		T	C	1.43 (35)			
rs61208773	155263166		C	A		1.85 (27)		1.42
rs4620533**	155262613		G	C	35.71 (35)	53.70 (27)	71.67 (90)	49.47
rs139885057	155262334		T	C	4.29 (35)	3.70 (27)		1.93
rs3762272**	155261777		G	A			66.84 (98)	26.62
rs1052176**	155260383	R569R	C	A	31.43 (35)	50.00 (27)	71.58 (95)	41.66
rs1052177**	155260350		T	C	31.43 (35)	50.00 (27)	71.58 (95)	47.62
rs932972**	155260096		C	T	31.43 (35)	50.00 (27)	71.58 (95)	48.61
rs8847**	155259323		G	A	30.88 (34)	50.00 (26)	71.50 (100)	46.45

All coding and non-coding *PKLR* variants identified within the Senegalese (total n = 62) and Thai (total n = 102; six from the exploratory cohort plus an additional 96 in the expanded Thai cohort) populations, with the alternative allele frequency (%) within the sequenced population. The total numbers of successfully sequenced samples are noted in parenthesis. Alternative allele frequencies registered within the dbSNP build 138 database (genome.ucsc.edu) are included for comparison. SNPs used to define haplotypes are marked with a “**”.

**Fig 2:**
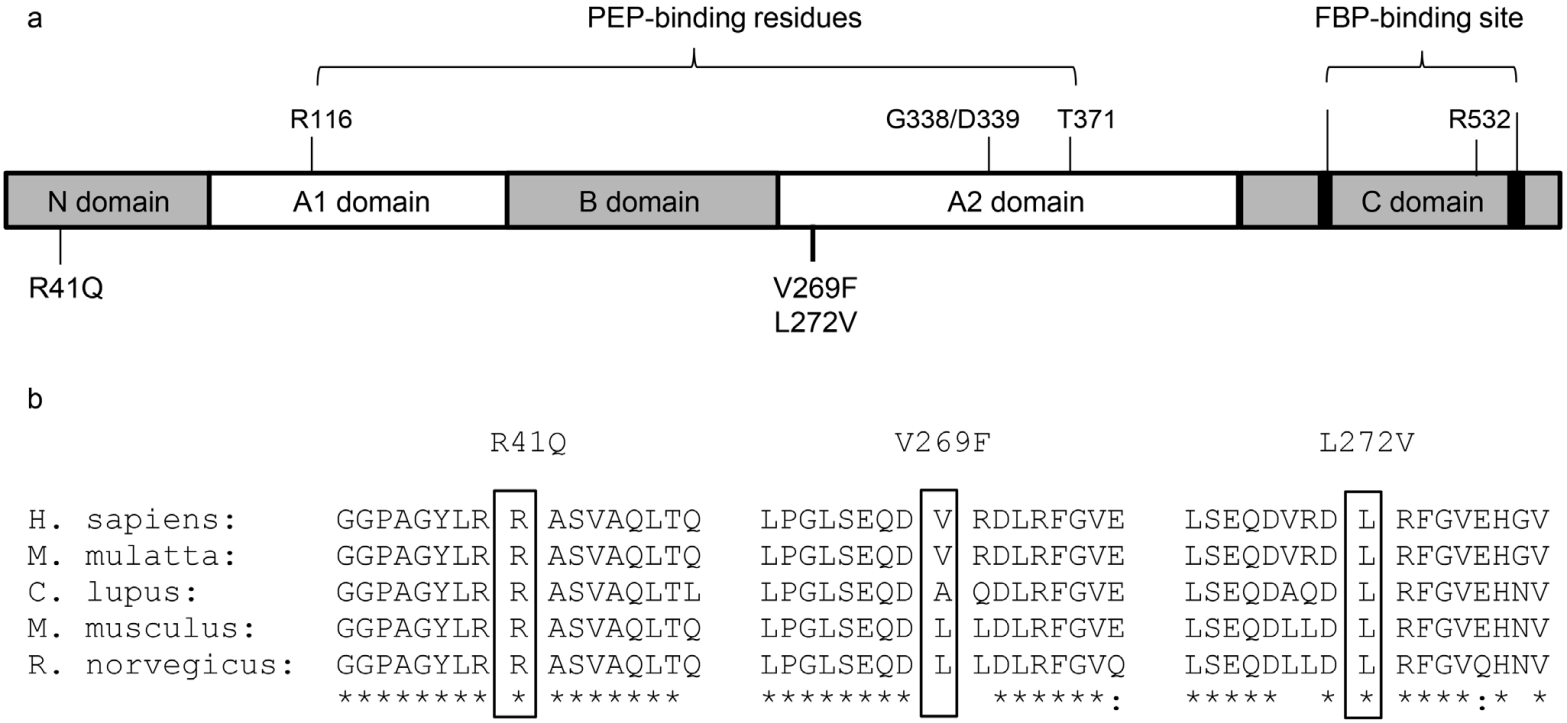
PKLR coding variants identified in the test populations. **(a)** Schematic representation of the PKLR protein, location of catalytically active residues (phosphoenolpyruvate, PEP, binding residues; R116, G338, D339, and T371) and allosteric regulation site (fructose-1,6-bisphosphate, FBP, binding site; loops 475–490 and 557–566, and residue R532) within structural domains [34, 46], and location of non-synonymous mutations identified from sequencing (R41Q, V269F and L272V). **(b)** Subset of multiple species protein alignment by Clustal Omega (www.ebi.ac.uk) where “*” = single, fully conserved residue; “:” = strongly similar properties

In the six Thai individuals sequenced, several common dbSNPs were present in addition to a novel non-synonymous mutation (c.161A>G) detected in a single individual (Table 1), resulting in a coding change from an arginine at residue 41 to glutamine (R41Q) (Fig 2). R41 is highly conserved across species and the arginine to glutamine substitution is not conservative and results in the loss of the positive side chain of wild-type R41. Sequencing an expanded cohort of 96 Thai individuals identified nine samples positive for R41Q (all heterozygotes), establishing an allele frequency of ~5% for this coding variant in the Thai population. Eleven additional variants with minor allele frequency > 1% were detected in the Thai cohort, including rs200695047 (c.844G>T) which codes for a valine to phenylalanine substitution at amino acid 269 (V269F). This mutation was identified in one homozygous and six heterozygous samples. V269 is not conserved throughout evolution (Fig 2) and the V269F homozygote did not display symptoms of PK-deficiency, suggesting that V269F does not affect PKLR protein activity. Haplotype analysis with informative SNPs (rs3020781, rs2071053, rs4620533, rs3762272, rs1052176, rs1052177, rs932972, rs8847) revealed 3 haplotypes in the Thai sample, with the most common haplotype (68.9%) being unique to the Thai community, and the two less frequent haplotypes being shared with the Senegalese samples (Fig 1). Interestingly, the R41Q variant was detected on the Thai-specific haplotype (Haplotype 1, Fig 1) suggesting that this mutation may be population-specific, having arisen on a Southeast Asian genetic background.

### Population genetic analysis of the R41Q and L272V variants in *PKLR*

Further characterization of possible associations between the L272V and R41Q variants and malarial phenotypes was undertaken, as these variants affect residues that are highly conserved in the PKLR protein family and may affect PKLR function. The L272V mutation (rs147659527) was identified in one homozygous individual from the Senegalese community of Ndiop. Further genotyping of the Ndiop community revealed that L272V was present in a single family, where the grandmother was homozygous, and three of her children and a grandchild were heterozygous. This family is one of 12 Fulani families in the Ndiop community (85 individuals), an ethnic group distributed throughout Western and Central Africa [18, 47, 48]. Additional sequencing of the *PKLR* gene in 149 unrelated individuals of Mossi (n = 89) and Fulani (n = 60) ethnicity from Burkina Faso identified three L272V heterozygotes in Mossi individuals. Hence, the L272V variant appears to be a rare variant (0.2%-1.5%; dbSNP build 138) distributed in different African populations. The relatively rarity of this variant in the African populations available was too low to examine possible association with haematological or malarial phenotypes.

Amongst the 102 Thai samples sequenced, we detected ten R41Q heterozygotes. Genotyping this mutation in the entire study population consisting of 871 samples from 181 families, and from 46 unrelated individuals identified one homozygote individual and 81 heterozygotes (71 of Karen origin), representing a mutant allele frequency of approximately 4.7% in this population. The R41Q genotypes exist in Hardy-Weinberg equilibrium (HWE) in this Thai sample set. The homozygous R41Q mutant individual did not exhibit extreme blood phenotypes, although its erythrocyte and reticulocyte counts were both low (falling within the lowest 10^th^ and 25^th^ percentile; data not shown). The predicted allele frequency of 5% may provide sufficient statistical power for association studies.

Genotyping for the R41Q mutation in an additional 340 unrelated Southeast Asian control samples, detected 13 R41Q heterozygotes, consisting of 2 Cambodians, 2 Laos, 2 Thais, 4 Myanmarese, and 3 Mons. The R41Q genotypes exist in HWE in this sample set with the allele frequency in each population, 1.5% in Cambodians, 1.8% in Laos, 1% in Thais (mixed ethnicity), 3.2% in Myanmarese, and 2.9% in Mon. It is interesting to note that the frequency of the R41Q allele is low in the general Thai population (~1%), while it is higher in the Thais of Karen ethnicity (~5%) who generally live in malaria-endemic areas. Similarly, the frequency of the R41Q allele is higher in the Myanmarese population (~3.2%), where a large population of ethnic Karen reside [49].

### Association studies

**Fig 3:**
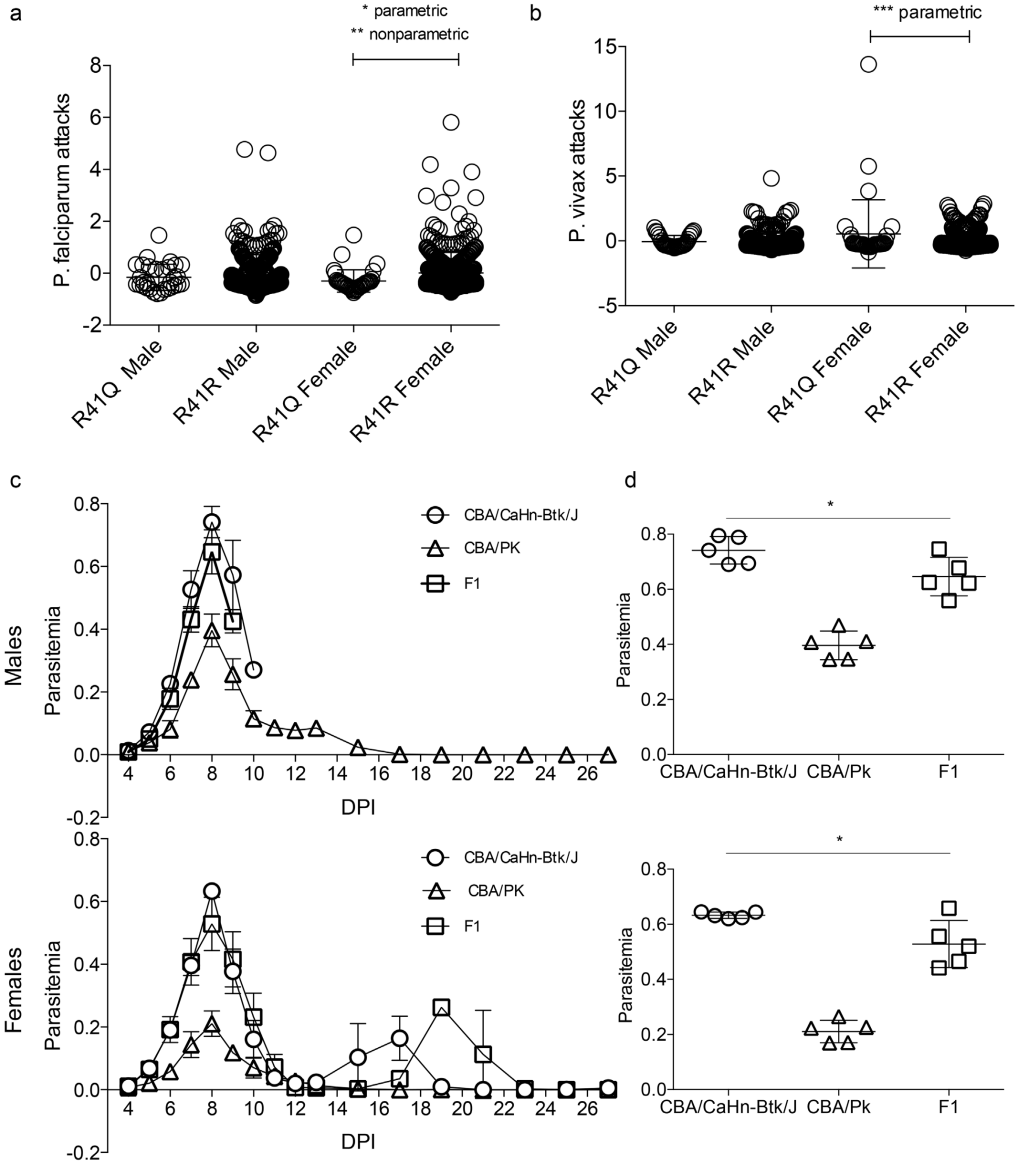
Effect of human and mouse PKLR variants on malaria phenotypes. **(a)** Association of the R41Q mutation and *Plasmodium falciparum* attacks in the genotyped Thai population. Residual *Plasmodium falciparum* attacks for heterozygous (R41Q) and homozygous wild-type (R41R) individuals. Both parametric and non-parametric tests indicate statistically significant decrease in attacks for heterozygous females only (ANOVA p = 0.0260 Kruskal-Wallis (KW) p = 0.0053). **(b)** Residual *Plasmodium vivax* attacks for heterozygous (R41Q) and homozygous wild-type (R41R) individuals. A statistically significant increase in attacks was noted in heterozygous females though only for the parametric test (ANOVA p = 0.0007), not the non-parametric test (KW p = 0.1789). **(c)** Time course of *P*. *chabaudi chabaudi* AS infection in PK-sufficient (CBA/CaHn-Btk^xid^/J, circles), PK-deficient (CBA/Pk^slc^, triangles) and F1 generation mice (squares). **(d)** Peak parasitemia (8dpi). Results are separated based on gender. Statistical significance was determined via unpaired, two-tailed t-tests, calculated by GraphPad (Prism) where “*” represents p<0.05. Statistically significant differences between PK-sufficient and F1 heterozygotes was noted for both genders (females: p = 0.0264, males: p = 0.0378).

Among the 871 Thai individuals genotyped for R41Q, one individual was homozygous for the mutation, 81 were heterozygous and 789 had the wild-type genotype. Associations between R41Q heterozygosity and various blood and malarial parameters listed in S3 Table and previously described [6], were examined. No association was detected between R41Q status and erythrocyte count, hematocrit, or reticulocyte levels and any other phenotypes except two. Indeed, a significant statistical association was noted between R41Q heterozygosity and a reduced frequency of *Plasmodium falciparum* attacks (mean frequency of heterozygotes (m_He_) = -0.23 ± 0.09 (n = 65) and mean frequency of homozygous wild-type (m_Ho_) = 0.01 ± 0.03 (n = 649); ANOVA p = 0.0097, Kruskal-Wallis (KW) p = 0.0022), with this association being significant only in females (m_He_ = -0.30 ± 0.11 (n = 34) and m_Ho_ = 0.01 ± 0.05 (n = 346); ANOVA p = 0.0260, KW p = 0.0053 versus m_He_ = -0.16 ± 0.13 (n = 31) and m_Ho_ = 0.02 ± 0.05 (n = 303) ANOVA p = 0.17, KW p = 0.1436 for males) (Fig 3A). Additionally, we determined that individuals heterozygous for R41Q are significantly more susceptible to *Plasmodium vivax* attacks than *Plasmodium falciparum* attacks (m_He_ = 0.49 ± 0.18 (n = 65) and m_Ho_ = -0.07 ± 0.04 (n = 649); ANOVA p = 1.32 10^−4^) again limited to females (m_He_ = 0.84 ± 0.28 (n = 34) and m_Ho_ = -0.07 ± 0.05 (n = 346), ANOVA p = 7.1 10^−5^) and not males (m_He_ = 0.10 ± 0.15 (n = 31) and m_Ho_ = -0.08 ± 0.06 (n = 303), ANOVA p = 0.31). This result strongly suggests that females and not males are differently susceptible to *Plasmodium falciparum* attacks and to *Plasmodium vivax* attacks depending on R41Q heterozygosity. Gender-specific effects for blood-stage malaria phenotypes have been previously reported in humans [11], and mice [45]. These findings suggest that heterozygosity for R41Q may have an impact on host susceptibility to infection with malarial parasites in endemic areas of disease.

### Heterozygosity for *Pklr* in mice protects against peak parasitemia

In humans and mice, pyruvate kinase deficiency is inherited in a strictly recessive fashion, with heterozygotes displaying no clinical symptoms, and displaying normal haematological profiles. Nevertheless, we have previously shown that *P*. *falciparum*-parasitized erythrocytes from subjects heterozygous for loss of function at *PKLR* are phagocytized more avidly than similarly infected wild-type erythrocytes [30, 33]. Hence, we tested the effect of heterozygosity for the known loss of function mutation at murine *Pklr* (G338D) [50], on haematological replication of the malarial parasite in a mouse model of infection with *P*. *chabaudi*. For this, wild-type controls (CBA/CaHn-Btk^xid^/J), pyruvate kinase deficient mutants (CBA/Pk^slc^) homozygous for a loss of function allele at *Pklr* (G338D), as well as F1 heterozygotes (G338D/+) were infected with *P*. *chabaudi* AS and blood parasitemia was followed over time (Fig 3C). We observed that male mice of all genotypes were more susceptible than female mice, in agreement with a previous report [45]. However, we noted that at the peak of infection (peak parasitemia), G338D/+ heterozygotes displayed lower peak parasitemia values than their wild-type counterparts (CBA/J), and this for both male (two-tailed unpaired t-test p = 0.0378) and female mice (p = 0.0264) (Fig 3D). In males, this significant difference in blood parasitemia between wild-type and G338/+ heterozygotes extended from day 7–9 post-infection (p = 0.0188; p = 0.0290). This suggests that heterozygosity for mutant *Pklr* variant in mice improves response to infection with the malarial parasite.

### R41Q recombinant protein exhibits modest effect on half-life

**Fig 4:**
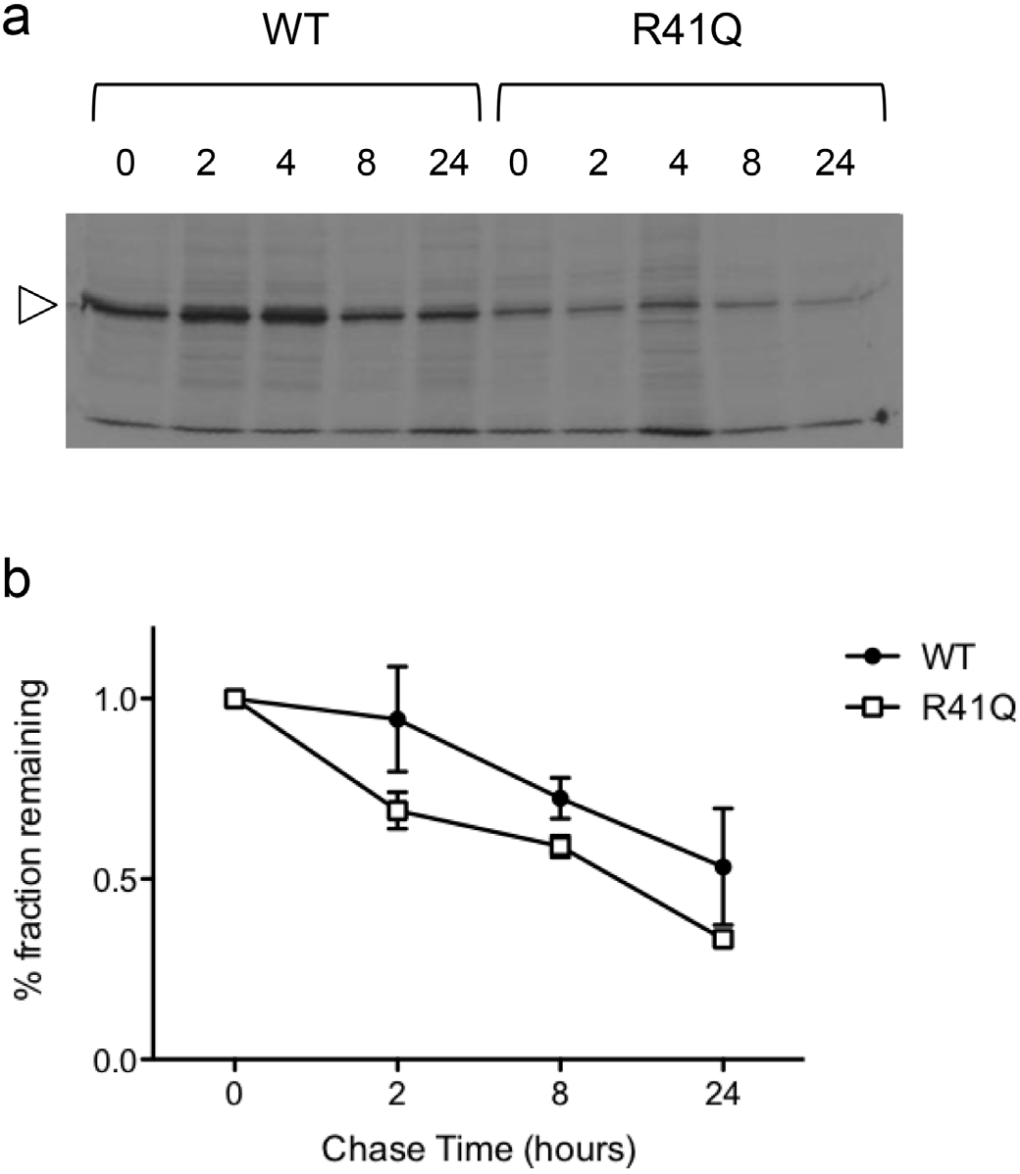
Effect of R41Q on PKLR protein stability. **(a)** Transiently transfected HEK293T cells expressing either the wild-type (WT) or the R41Q mutant PKLR proteins were metabolically labelled with [^35^S]-methionine followed by chase in label-free media for the indicated time period (in hours). Cell lysates were prepared and PKLR was immunoprecipitated, followed by gel electrophoresis. **(b)** The radiolabelled PKLR species were quantitated using ImageJ software, and averages from 2–3 independent experiments were calculated as a fraction of the maximum labelling observed at 0 hr.

Overexpression of the R41Q variant in transiently transfected HEK293T cells, followed by semi-quantitative measurement of pyruvate kinase activity (PK-LDH assay) failed to detect a major effect of the R41Q mutation on basal enzymatic activity in such over-expressing cells (data not shown). We also examined the effect of the R41Q mutation on stability of the transiently transfected [^35^S]-methionine labelled protein in pulse-chase studies (Fig 4). In three independent experiments, we observed a shorter half-life for R41Q compared to WT protein expressed in transfected cells: The effect was modest but reproducible, suggesting that R41Q may affect protein stability in these cells.

## Discussion

Malaria kills one child every minute in Sub-Saharan Africa [51, 52]. Over time, selective pressure from such a lethal infection has resulted in the genetic retention in malaria endemic areas of certain allelic variants that confer partial protection against disease. The erythrocyte is the major replication niche of the *Plasmodium* parasite, and variants in certain erythrocyte proteins have been found enriched in malaria-endemic areas. Although homozygosity for such variants causes disease on their own (sickle cell anemia, thalassemias, G6PD deficiency), heterozygosity confers various degrees of protection against malaria [1–3, 53, 54]. We have previously shown that homozygosity for loss of function mutations within the erythrocyte pyruvate kinase gene (*Pklr*) in mice reduces the severity of infection with the blood stage parasite *P*. *chabaudi* AS [31, 32]. Likewise, in human erythrocytes infected *ex vivo* with *P*. *falciparum*, homozygosity for PK-deficiency alleles causes a reduction in parasite replication, while parasitized erythrocytes from heterozygotes are phagocytized more avidly than those from normal individuals [30, 33]. Using comparative analyses of archival populations contained in the CEPH Human Diversity panel from current and ancestral areas of malaria, we detected rich genetic diversity in the *PKLR* gene, including the presence of rare, potential loss-of-function protein variants. Neutrality testing of *PKLR* haplotypes and variants suggested positive selection of the gene in the sub-Saharan African and Pakistani populations, possibly by the malarial parasite [34]. This hypothesis remained to be formally tested, and was the major aim of the current study.

Herein, we probed for the possible association between rare or common variants at the sequenced *PKLR* gene, and haematological and malaria-associated phenotypes in three populations from Thailand and Senegal, living in areas of seasonal or year-long exposure to the malarial parasite. These populations were studied longitudinally for multiple phenotypes, including but not limited to frequency and intensity of infection by the malarial parasites, thereby providing added power to detect genetic effects of the host in response to the parasite. The Thai population has been used in the past to document the effect of G6PD deficiency (*Mahidol* allele) on differential susceptibility to infection with *P*. *falciparum* and *P*. *vivax* [11].

Sequencing the *PKLR* gene in subsets of Senegalese and Thai individuals identified a number of common variants (SNPs), nine of which were used to define *PKLR* haplotypes in these populations. Eight *PKLR* haplotypes were detected, two of which were common and representing approximately 86% of the Senegalese and 31% of the Thai populations. These common haplotypes are also present in various North, South, and Sub-Saharan African, Pakistani, Southeast Asian and European populations [34]. Haplotype analysis at *PKLR* further detected population substructure in the two Senegalese populations. The Dielmo community is composed primarily of the Serere and Mandinka ethnic groups [7], while the Ndiop community is comprised predominantly of Wolof and Fulani ethnicities [7]: unique *PKLR* haplotypes were detected in these populations. Of particular interest is the Thai-specific haplotype 1 (Fig 1) onto which the R41Q mutation appeared to have emerged. This strongly suggests that the R41Q variant is population specific. We also detected the R41Q variant in Cambodian, Lao, Myanmarese, and Mon populations from other malaria-endemic areas. Although malaria-associated phenotypes are not available for these populations, presence of the R41Q mutation suggests that the variant occurred on an ancient South-Asian haplotype which is shared amongst populations of Thailand, Laos, and Cambodia.

Sequencing also identified three coding variants in the PKLR protein; these variants affected residues that were either highly conserved (R41, L272), or coded for a very significant change in amino acid property (V269F), both of which possibly result in altered protein function. The L272V and V269F variants were present at very low frequency in the two populations, and association with haematological and malaria traits could not be conducted with sufficient statistical power. Genotyping revealed that the R41Q mutation was present with a minor allele frequency of approximately 4.5% within the Thai cohort. We noted a statistically significant association between R41Q heterozygosity and a reduced frequency of *Plasmodium falciparum* attacks, with this association being significant only in females. Additionally, there appears to be a tendency for R41Q heterozygotes to be more susceptible to infection with *Plasmodium vivax*, although this was only significant in parametric, rather than non-parametric tests, and again limited to females (Fig 3A and 3B). Although these findings require replication in additional cohorts of individuals with informative R41Q genotypes and from malaria-endemic areas, the current results suggest a possible association of the R41Q variant with reduced susceptibility to *P*. *falciparum* infection. The increased susceptibility to *P*. *vivax* infection noted in some R41Q heterozygotes and concomitant to reduced susceptibility to *P*. *falciparum* may reflect immunological cross-reactivity and immune protection in regions of mixed infections [55]. On the other hand, such genetically influenced differential susceptibility to different malarial parasites has been previously reported in the case of glucose-6-phosphate dehydrogenase deficiency. Indeed, it was demonstrated that the G6PD^*Mahidol*^ (487A) variant reduces infection with *P*. *vivax*, but not with *P*. *falciparum* in a population from South Asia, suggesting that *Plasmodium vivax* has been a driving force behind the strong selective advantage conferred by this mutation [11].

Pyruvate kinase is composed of an amino terminal (N) domain with a helix-turn-helix motif, followed by a bi-partite A domain (A1, A2) that contains residues implicated in binding the phosphoenolpyruvate (PEP) substrate, as well as a conserved B domain linker, followed by the C-terminal domain that binds the allosteric regulator fructose-1,6-bisphosphate (FBP) (Fig 2). Structure function studies have shown that PKLR exists either in the active R-state or the inactive T-state, and that the state of activation is regulated by intra- and inter-domain interactions between the A and C domains of adjacent subunits as well as the A/B and A/C domains within a subunit [26, 56, 57]. The R41Q mutation is located within the N-terminal domain of PKLR, and the arginine residue is highly conserved in mammals. While overexpression of the R41Q variant in transfected cells failed to identify a significant effect of the mutation on enzymatic activity, we noted a modest but reproducible effect of R41Q on protein half-life in pulse-chase studies.

In addition to basal activity, there are number of structural and functional aspects of PKLR that may be affected by R41Q. PKLR activity is regulated in part by phosphorylation of serine 43, which causes increased affinity for allosteric inhibitors ATP and alanine, and decreased affinity for PEP and the allosteric activator FBP [58, 59]. Two mutations mapping to the N-terminal domain of PKLR (near R41Q) have been functionally characterized. One mutation (A36G) identified in a case of congenital nonspherocytic hemolytic anemia with PK-deficiency, was shown to partially impair enzyme activity [60]. A possible gain-of-function variant (G37E) was found associated with enzyme hyper-activity, increased ATP levels, and possibly linked to altered phosphorylation of S43 [61, 62]. Additionally, the PK-R isoform undergoes post-translational proteolytic cleavage of the N-terminus during erythrocyte maturation, generating full length and truncated proteins with different capacity for homo- and heterotetramerization [24, 26, 46, 57, 63]. Also, fructose-1,6-bisphosphate promotes tetramer formation, while ATP causes tetramer disassociation (PK-R/L, M2) [64, 65]. Together, these findings suggest that the N-terminal domain of PKLR that carries the R41Q variant plays a key role in enzyme function, regulation of activity and functional oligomerization. Finally, PKLR monomers can oligomerize with monomers from the other isoform, PK-M2, leading to the formation of hybrids that contain 1 to 3 subunits of either isoform [66–69]. PK-M2 is broadly expressed in most cells lines used in transfection studies, including HEK-293 cells used herein, which further complicates the functional study of PKLR variants expressed in these cells. Indeed, it is possible that R41Q affects the ability of the PK-R enzyme to heterodimerize with background PK-M2 proteins present in transfected cells, leading to PK multimers of unknown activity.

Finally, it has been observed that some disease-associated *PKLR* mutations do not affect enzymatic activity of the purified protein. For example, the R510Q mutant in a patient with nonspherocytic hemolytic anemia shows wild-type kinetic activity and regulatory properties when expressed in bacteria [57]: However, R510Q exhibits significantly lower thermal stability when compared to the wild-type control, suggesting that protein instability rather than reduced enzymatic activity is responsible for PK-deficiency in R510Q patients [57]. Additional studies will be required to characterize the molecular effect of the R41Q variant on PKLR structure and function.

Although our study requires additional validation in other cohorts where longitudinal data on haematological malaria phenotypes are available, results obtained so far strongly suggest that allelic variants at PKLR modulate susceptibility to *Plasmodium* infection in humans, with protective alleles possibly retained in populations living in areas of endemic malaria. The cumulative data supporting this proposal include: a) the demonstration that homozygosity for loss of function at *Pklr* cause complete protection against blood-stage malaria (*P*. *chabaudi AS*) in mice [31, 32], with heterozygotes showing reduced levels of blood-stage parasitemia (Fig 3); b) homozygosity and heterozygosity for human PK-deficiency associated loss of function alleles at *PKLR* cause a reduction in parasite replication and increase phagocytosis of parasitized erythrocytes in studies with primary erythrocytes infected *ex vivo* with *P*. *falciparum* [30]; c) the *PKLR* gene shows highest sequence diversity in regions where malaria is or has been endemic, when compared to regions where it has not been prevalent [34]; d) a unique PKLR variant (R41Q) with reduced protein stability has appeared on a common haplotype from Southeast Asia and shows an association with reduced number of malaria attacks in females from a large Thai cohort of Karen ethnicity from a malaria endemic area. Therefore, along with haemoglobin, glucose-6-phosphate dehydrogenase, anion exchanger band 3, and several others, PKLR would represent another example of an erythrocyte-specific protein in which heterozygosity for partial or complete loss of function variants confer some degree of protection against malaria.

## Supporting Information

S1 Table Sequencing primers designed to span the exon and intron/exon junctions of the *PKLR* gene. Adapted from Berghout *et*. *al*. [34].
(DOCX)

S2 Table Main characteristic of the Thai cohort (n = 897) investigated in this study. (DOCX)

S3 Table List of haematological and malaria phenotypes investigated in this study. (DOCX)

S4 Table Site directed mutagenesis primers. Primers were designed to have at least 10 nucleotides flanking the single point mutation (in bold).
(DOCX)

S5 Table Construct sequencing primers. Designed within the PKLR cDNA insert and pcDNA3 vector, these primers were utilized in confirming the sequence of the wild-type and mutant constructs.
(DOCX)
