# Supplementary material for: Modulation of Malaria Phenotypes by Pyruvate Kinase (PKLR) Variants in a Thai Population
Source: PLoS One. 2015 Dec 14;10(12):e0144555. doi: 10.1371/journal.pone.0144555 (PMC4677815; doi:10.1371/journal.pone.0144555)
Supplement: S1 Table — Adapted from Berghout et. al. [34]. (DOCX) [file pone.0144555.s001.docx]

| Primer name | Primer sequence (5'-3') |
| --- | --- |
| Exon11c-F | 5'-GGC AAA TGG CTG AAT CCT TG |
| Exon11c-R | 5'-ATA ACC CCA GGC ATC CCA TC |
| Exon11a-F | 5'-GCA CGA GAT GCC CAG ATT AT |
| Exon11a-R | 5'-GCC TGG TCC CTC AAT GAC TA |
| Exon10-F | 5'-AAG CAC ATA ATG GAC TTT CTC AAA |
| Exon10-R | 5'-TGG CAG GGA AGG TCT AGG TA |
| Exon8,9-F | 5'-TTT CAG GGG TTG TGA CTG TGA |
| Exon8,9-R | 5'-TCC TGT TAA TCC TGC CAA CC |
| Exon6,7-F | 5'-AAA CCC ACA GAG TGC CGA AC |
| Exon6,7-R | 5'-AGC CCT CAG ACC GAT CAC AC |
| Exon3,4,5-F | 5'-GTG AGG CGT TCT GAG AAT GGT |
| Exon3,4,5-R | 5'-GAA GGT GTG ATC GGT CTG AGG |
| Exon2-F | 5'-ACT GGG TGA TTC TGG GTC TG |
| Exon2-R | 5'-CCC AAG GGT AGG GAT TTT TG |
| Exon1b-F | 5'-CAC CCA GTT CCT GCT CCA AA |
| Exon1b-R | 5'-TAC TGG GTG TGC CCC TTT TC |
