# Supplementary material for: Modulation of Malaria Phenotypes by Pyruvate Kinase (PKLR) Variants in a Thai Population
Source: PLoS One. 2015 Dec 14;10(12):e0144555. doi: 10.1371/journal.pone.0144555 (PMC4677815; doi:10.1371/journal.pone.0144555)
Supplement: S2 Table — (DOCX) [file pone.0144555.s002.docx]

| Age Mean ± SE (min, max) | Nb of Male | Nb of Female | Nb PFA Mean ± SE (min,max) | Nb PVA Mean ± SE (min,max) |
| --- | --- | --- | --- | --- |
| 23.33 ± 12.93 (1.18,75.92) | 415 | 482 | 1.22 ± 1.79 (0, 12) | 0.59 ± 1.30 (0, 13) |
