# Supplementary material for: Modulation of Malaria Phenotypes by Pyruvate Kinase (PKLR) Variants in a Thai Population
Source: PLoS One. 2015 Dec 14;10(12):e0144555. doi: 10.1371/journal.pone.0144555 (PMC4677815; doi:10.1371/journal.pone.0144555)
Supplement: S3 Table — (DOCX) [file pone.0144555.s003.docx]

| **Phenotype** | **Acronym** |
| --- | --- |
| ***A. Clinical Phenotypes*** |  |
| Number of Pf attacks | PFA |
| Proportion of Pf attacks | PPFA |
| Number of non-malaria fever visits | NMF |
| Number of Pv attacks | PVA |
| Proportion of Pv attacks | PPVA |
| ***B. Parasite Biological Phenotypes*** |  |
| Pf max parasite density | mx-PFD |
| Pf parasite density | PFD |
| Pv max parasite density | mx-PVD |
| Pv parasite density | PVD |
| ***C. Blood cell count Phenotypes*** |  |
| Hemoglobin A0 | A0result |
| Hemoglobin A1 | A1result |
| Hemoglobin A2 | A2result |
| Hemoglobin E | Eresult |
| Hemoglobin F | Fresult |
| White blood cell count | WBC |
| Red blood cell count | RBC |
| Hemoglobin | HGB |
| Hematocrit | HCT |
| Mean corpuscular volume | MCV |
| Mean corpuscular hemoglobin | MCH |
| Mean corpuscular hemoglobin concentration | MCHC |
| Platelet count | PLT |
| Red blood cell distribution width Standard Deviation | RDW-SD |
| Red blood cell distribution width CV | RDW-CV |
| Platelet distribution width | PDW |
| Mean platelet volume | MPV |
| Platelet larger cell ratio | P-LCR |
| Procalcitonin blood test | PCT |
| Neutrophil count | NEUT |
| Percentage of neutrophils | PNEUT |
| Lymphocyte count | LYMPH |
| Percentage of lymphocytes | PLYMPH |
| Monocyte count | MONO |
| Percentage of monocytes | PMONO |
| Eosinophil count | EO |
| Percentage of eosinophils | PEO |
| Basophil count | BASO |
| Percentage of basophils | PBASO |
| nucleated red blood cell count | NRBC |
| Percentage of nucleated red blood cells | P100NRBC |
| Reticulocyte count (Percentage) | RET |
| Reticulocyte count | TetaRET |
| Immature reticulocyte fraction | IRF |
| Low fluorescence of auramine bound to rRNA | LFR |
| Medium fluorescence of auramine bound to rRNA | MFR |
| High fluorescence of auramine bound to rRNA | HFR |
