# Supplementary material for: Modulation of Malaria Phenotypes by Pyruvate Kinase (PKLR) Variants in a Thai Population
Source: PLoS One. 2015 Dec 14;10(12):e0144555. doi: 10.1371/journal.pone.0144555 (PMC4677815; doi:10.1371/journal.pone.0144555)
Supplement: S4 Table — Primers were designed to have at least 10 nucleotides flanking the single point mutation (in bold). (DOCX) [file pone.0144555.s004.docx]

| Primer name | Primer sequence (5'-3') |
| --- | --- |
| R41Q forward | 5'-ATC TGC GGC **A**GG CCA GTG T |
| R41Q reverse | 5'-ACA CTG GCC **T**GC CGC AGA T |
| D390N forward | 5'-AGA GAC AAG C**A**A TGT CGC CAA |
| D390N reverse | 5'-TTG GCG ACA T**T**G CTT GTC TCT |
