# Supplementary material for: Modulation of Malaria Phenotypes by Pyruvate Kinase (PKLR) Variants in a Thai Population
Source: PLoS One. 2015 Dec 14;10(12):e0144555. doi: 10.1371/journal.pone.0144555 (PMC4677815; doi:10.1371/journal.pone.0144555)
Supplement: S5 Table — Designed within the PKLR cDNA insert and pcDNA3 vector, these primers were utilized in confirming the sequence of the wild-type and mutant constructs. (DOCX) [file pone.0144555.s005.docx]

| Primer name | Primer sequence (5'-3') |
| --- | --- |
| pcDNA3F | 5'-CGG TGG GAG GTC TAT ATA AG |
| pcDNA3R | 5'-AGG AAA GGA CAG TGG GAG TG |
| R41Qright | 5'-ACC CGG ACA ATA TTG GGG TA |
| R41Qleft | 5'-GCT TCG GTC ATG GGT CTC TA |
| L272Vright | 5'-TCT TCT GAG CCA GGA AAA CC |
| L272Vleft | 5'-CGA GTA CCA TGC TGA GTC CA |
| D390Nleft | 5'-CAC GGC ATC AAG ATC ATC AG |
| D390Nright | 5'-GGA GGT TCA CGG TAA AGC AA |
| INSERTleft | 5'-ACT AAG CCG TGA TCC CAC TG |
| INSERTright | 5'-GGC CTT GAT CAT CTC CTT G |
